# S120. FACTORS ASSOCIATED WITH SUICIDE ATTEMPTS AMONG PATIENTS WITH SCHIZOPHRENIA

**DOI:** 10.1093/schbul/sby018.907

**Published:** 2018-04-01

**Authors:** Rahma Nefzi, Amine Larnaout, Hanen Ben Ammar, Emira Khelifa, Amina Aissa, Zouhaier EL Hechmi

**Affiliations:** Razi Hospital

## Abstract

**Background:**

Suicide is the leading cause of surmortality among patients with schizophrenia.

Despite efficient antipsychotic treatments, suicide rates reaches up to 15% of death causes and near half of the patients had at least once attempted suicide. Thereby, the early identification of clinical profiles and risk factors is important for the development of management strategies.

**Methods:**

A cross-sectional and retrospective descriptive study was conducted at the “F” psychiatry department at the Razi Hospital, Manouba including 56 patients with schizophrenia in period of clinical stability. The evaluation focused on sociodemographic and clinical characteristics (using the positive and negative syndrome scale (PANSS); The Calgary Depression Scale for Schizophrenia (CDSS); The Global Assessment of Functioning (GAF); The Clinical Global Impression (CGI) rating scales). Personal history of suicidal attempts was assessed.

**Results:**

In this study, fourteen patients with schizophrenia (25%) never attempted suicide. 58 % (N=32) committed one or two suicide attempts. Only 8.5% (N= 5) had 3 and 4 attempts each. Number of suicide attempts was negatively correlated with the age of onset (p=0.024, r=-0.442) and the GAF score (p= 0.002, r=- 0.483). An association was found between the personal history of suicidal attempts and the existence of a triggering factor of the onset (p=0.03). A positive correlation was found with the number of hospitalization (p=0.14, r=0.663), with the PANSS items: delusions (p=0.41, r=0.358), hallucinations (p=0.12, r=0.402), Suspiciousness/persecution (p=0.35, r= 0.342) and Somatic concern (p=0.048, r=0.322); with the CDSS guilty ideas of references (p=0.008, r=0.426) and with the CGI efficacy index (p=0.32, r=0.348).

**Discussion:**

The rates of suicide are the highest among patients with schizophrenia. Previous studies have estimated the prevalence of suicide attempts in individuals with schizophrenia up to 50%.

The increase in suicide attempts is associated with depressive symptoms which are very common within schizophrenia. The risk of suicide is not constant during the evolution of schizophrenia: it is the highest during the first years. A meta-analysis of 29 studies (Hawton et al., 2005) related that risk factors for suicide was higher in schizophrenic Caucasian men, those who live alone, who have recently experienced a loss, who have a family history of depression, who are more educated and have a higher IQ. Alteration of the abnormality the serotonin system may provide a biologic base to this phenomenon. It is essential to try to early detect and carefully assess the demographic and clinical profiles of patients with high risk of suicide.

